# Mitochondrial genome of the Antarctic microalga *Micractinium simplicissimum* KSF0127 (Chlorellaceae, Trebouxiophyceae)

**DOI:** 10.1080/23802359.2021.1886010

**Published:** 2021-03-15

**Authors:** Eun Jae Kim, Hyunsik Chae, Jihyeon Yu, Hyunjoong Kim, Sung Mi Cho, Seung Chul Shin, Han-Gu Choi, Sanghee Kim, Se Jong Han

**Affiliations:** a Division of Life Sciences, Korea Polar Research Institute, Incheon, South Korea; b School of Life Sciences, Kyungpook National University, Daegu, South Korea; c Department of Systems Biology, Institute of Life Science and Biotechnology, Yonsei University, Seoul, South Korea; d Department of Polar Sciences, University of Science and Technology, Incheon, South Korea

**Keywords:** *Micractinium simplicissimum*, Chlorellaceae, Trebouxiophyceae, Antarctic microalga, mitochondrial genome

## Abstract

We report the first mitochondrial genome of the Antarctic microalga *Micractinium simplicissimum* KSF0127. The circular mitochondrial genome was 67,923 bp in length and contained 45 protein-coding genes, one ribosomal RNA gene, and 60 transfer RNA genes. The phylogenetic tree was constructed with eight previously reported mitogenome sequences and showed the phylogenetic position of *M. simplicissimum* KSF0127 within the Chlorellaceae family.

Antarctica has a very cold and harsh environment, but various microalgae live by adapting to the extreme conditions. New Antarctic psychrotrophic and psychrophilic microalgae are being discovered, and evolutionary research using the mitochondrial genome is being conducted. *Micractinium* is a green microalga in the Chlorellaceae family that is characterized by spherical or ovoid cells. Recently, the Antarctic *M. simplicissimum* KSF0127 was identified as a new species by Chae et al. (2019). In this study, the mitochondrial genome of *M. simplicissimum* KSF0127 was reported for the first time. *M. simplicissimum* KSF0127 was isolated from freshwater in Robert Island, South Shetland Islands, Antarctica (62°23′02.80″ S, 59°41′31.50″ W) and deposited in the Korea Polar Research Institute (KOPRI) Culture Collection (https://pamc.kopri.re.kr). The culture was grown in freshwater solution BG-11 (Sigma-Aldrich, St. Louis, MO) at 2 °C under 25 μmol photons m^−2^ s^−1^ (16:8 h light:dark cycle) for 4 weeks. Total genomic DNA of *M. simplicissimum* KSF0127 was extracted using the i-genomic Plant DNA Extraction Kit (iNtRON Biotechnology, Seongnam, Korea), followed by library preparation using a TruSeq Nano DNA Kit (Illumina, San Diego, CA). Whole genome sequencing was performed using the HiSeq 4000 platform (Macrogen, Seoul, Korea) and raw data were filtered to obtain >9 Gb high-quality reads. De novo genome assembly was performed using the De Bruijn graph assembler and FALCON, and Prokka v.1.12b was used for gene prediction and annotation. Thus, the 67,923-bp long circular mitochondrial genome of *M. simplicissimum* KSF0127 was obtained (GenBank accession number: MW125581), and the nucleotide composition was 35.6% A, 35.2% T, 14.3% G, and 14.5% C. The overall GC content was 28.8%. This content was slightly higher than that of *M. singularis* MM0003 (GC content, 27.5%) and slightly lower than that of *M. pusillum* (GC content, 31.3%). *M. simplicissimum* KSF0127 mitogenome contains 45 protein-coding genes, one ribosomal RNA gene, and 60 transfer RNA genes. A phylogenetic tree was constructed using MEGA7 (Kumar et al. 2016) with eight previously reported mitochondrial genomes with 10 protein-amino acid sequence (COX1, 2, 3 and NADH1, 2, 3, 4, 5, 6, 7) belonging to the Chlorellaceae family, and the maximum-likelihood method with 1000 bootstrap replicates was used for analysis (Fan et al. 2017). The results showed the phylogenetic placement of *M. simplicissimum* KSF0127 within Chlorellaceae (Figure 1). *M. simplicissimum* KSF0127 used in the phylogenetic tree was isolated from fresh water in Robert Island, Antarctica, *M. pusillum* CCAP 231/1 was isolated from fresh water in Cambridgeshire, Wicken Lode, UK. *Chlorella* sp. ArM0029B was isolated from marine water at Arctic Kongsfjorden, *M. singularis* MM0003 was isolated from marine water at Janghang Harbor, Korea, and *M. conductrix* was isolated from *Paramecium bursaria*, a species of Ciliate. The regional distribution and sources of *M. simplicissimum* KSF0127 and related species reported to date were diverse. In the previous study, three *Micractinium* species (*M. pusillum* CCAP 231/1, *M. singularis* MM0003, *M. conductrix*) were reported to have a close relationship with *Chlorella* sp. ArM0029B within Trebouxiophyceae (Jo et al. 2020; Kang et al. 2020). In addition, our study also showed that *M. simplicissimum* KSF0127 has the close relationship with *Chlorella* sp. ArM0029B within the *Micractinium* branch. This new mitogenome sequence could be useful for evolutionary studies on phylogenetic relationships among *Micractinium* species, including Antarctic species of the Chlorellaceae family.

**Figure 1.**
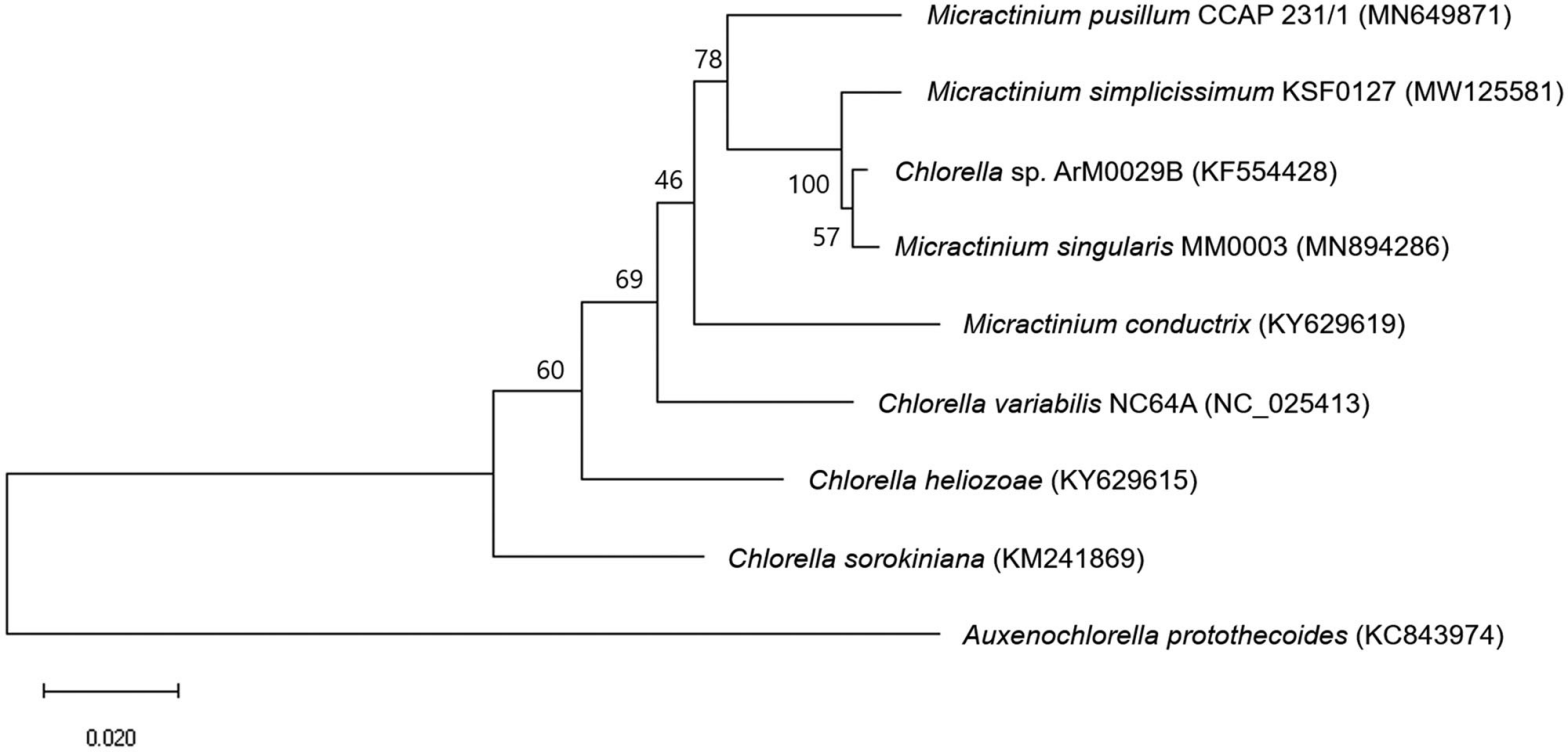
Maximum-likelihood phylogenetic tree of *M. simplicissimum* KSF0127 and eight other species. The numbers listed on each node represent the bootstrap support value. GenBank accession numbers were indicated in the parentheses.

## Data Availability

The data that support the findings of this study are openly available in GenBank of NCBI at https://www.ncbi.nlm.nih.gov/ under the accession number MW125581. The associated BioProject and BioSample number are PRJNA681449 and SAMN16954049, respectively.
